# Association of Maternal Immunity with Rotavirus Vaccine Immunogenicity in Zambian Infants

**DOI:** 10.1371/journal.pone.0150100

**Published:** 2016-03-14

**Authors:** Roma Chilengi, Michelo Simuyandi, Lauren Beach, Katayi Mwila, Sylvia Becker-Dreps, Devy M. Emperador, Daniel E. Velasquez, Samuel Bosomprah, Baoming Jiang

**Affiliations:** 1 Centre for Infectious Disease Research in Zambia, Lusaka, Zambia; 2 University of North Carolina at Chapel Hill, Chapel Hill, North Carolina, United States of America; 3 Centres for Disease Control and Prevention, Atlanta, Georgia, United States of America; University of Nebraska-Lincoln, UNITED STATES

## Abstract

**Introduction:**

Live attenuated oral vaccines against rotavirus (RV) have been shown to be less efficacious in children from developing countries. Reasons for this disparity are not fully understood. We assessed the role of maternal factors including breast milk RV-specific IgA, transplacentally acquired infant serum RV-specific IgG and maternal HIV status in seroconversion among Zambian infants routinely immunized with Rotarix™ (RV1).

**Methods:**

420 mother-child pairs were recruited at infant age 6–12 weeks in Lusaka. Clinical information and samples were collected at baseline and at one month following the second dose of RV1. Determination of breast milk RV-specific IgA and serum RV-specific IgA and IgG was done using standardized ELISA. Seroconversion was defined as a ≥ 4 fold rise in serum IgA titre from baseline to one-month post RV1 dose 2, while seropositivity of IgA was defined as serum titre ≥ 40 and antibody variables were modelled on log-base 2. Logistic regression was used to identify predictors of the odds of seroconversion.

**Results:**

Baseline infant seropositivity was 25.5% (91/357). The seroconversion frequency was 60.2% (130/216). Infants who were IgA seropositive at baseline were less likely to seroconvert compared to their seronegative counterparts (*P* = 0.04). There was no evidence of an association between maternal HIV status and seroconversion (*P* = 0.25). Higher titres of breast milk rotavirus-specific IgA were associated with a lower frequency of seroconverson (Nonparametric test for trend Z = -2.84; P<0.01): a two-fold increase in breast milk RV-specific IgA titres was associated with a 22% lower odds of seroconversion (OR = 0.80; 95% CI = 0.68–0.94; P = 0.01). There was seasonal variation in baseline breast milk rotavirus-specific IgA titres, with significantly higher GMTs during the cold dry months (*P* = 0.01).

**Conclusion:**

Low immunogenicity of RV1 vaccine could be explained in part by exposure to high antibody titres in breast milk and early exposure to wild-type rotavirus infections. Potential interference of anti-RV specific IgA in breast milk and pre-vaccination serum RV specific-IgA and IgG titres with RV1 seroconversion and effectiveness requires further research.

## Introduction

Rotavirus (RV) kills half a million children each year worldwide including approximately 230,000 children in sub-Saharan Africa, making it the most significant contributor to diarrhoea-related child deaths worldwide [1–3]. RV is a vaccine-preventable illness. The demonstrated effectiveness of RV vaccines, however, is generally low and highly variable in developing countries [4,5]. Vaccines are among the most cost effective tools available to prevent infectious diseases, and international guidelines recommend widespread roll-out of RV vaccine [6,7]. Rotarix™ (GlaxoSmithKline Biologicals, Rixensart, Belgium), a monovalent (RV1) attenuated human oral vaccine based on the G1P[8] RV strain [8], was recently introduced in the routine immunisation programme in Zambia. Its safety and efficacy have been established in healthy infants across multiple continents, including Africa [9–12]. However, the vaccine has been found less efficacious among children in low income settings as compared with middle income and industrialized countries for reasons not yet completely understood [13–15]. Indeed, in a recent multicentre trial of RV1 conducted in South Africa and Malawi, the vaccine showed substantially better efficacy in South Africa (76.9% efficacy) than in Malawi (49.4% efficacy) [16].

It has been proposed that high background rates of malnutrition; other enteric co-infections; chronic diseases such as HIV, TB and recurrent malaria; co-administration of RV vaccine with OPV; and interference from passively acquired maternal antibody may all play a role in this reduced vaccine responsiveness [17–22]. Other reports have investigated the potential inhibitory role of acquired and innate immunological components of breast milk, which have provided suggestive *in-vitro* evidence [21,23]. Lastly, rotavirus natural infection and disease has been known to be high during the cool dry seasons, and indeed some reports have documented marked seasonal variations in rotavirus mortality and vaccine efficacy in children following national introduction of vaccines [13, 16]. In Zambia, seasonal trends for rotavirus infection have also been reported [24–26].

We set out to evaluate the possible contribution of maternal RV-specific immunity factors on failed infant seroconversion following routine RV vaccination in a cohort of 420 Zambian mother-infant pairs. Here we present findings exploring associations of maternal factors and seasonality, on infant seroconversion to RV immunisation.

## Materials and Methods

### Study site and participants

The study was conducted at Kamwala clinic, a peri-urban primary care health facility in Lusaka, which provides basic outpatient and maternal child health services. Kamwala has a catchment population of over 10,000 with approximately 120 new infants presenting each month. The majority of the attendees to this public facility are low-income residents of two nearby sprawling shanty compounds.

Study recruitment was done between April 2013 and March 2014. We recruited participants as they presented to the facility for routine immunisation service. Mother-infant pairs were approached during the initial visit with study information. Those interested were provided detailed information. Consenting mothers were administered a simple comprehension text and those meeting the minimum understanding provided individual written consent. Mothers unable to read or write were provided information through an impartial witness who provided a signed confirmation together with thumbprint of the participant as confirmed written consent. The pair was eligible if: i) the mother was willing to participate voluntarily and able to provide signed informed consent (with witness in the case of illiterate participant); ii) the infant was eligible for RV1 vaccine immunization as per national policy (male or female infant, aged 6–12 weeks old); iii) the mother was willing to undergo study procedures, including questionnaires, HIV counselling and testing, CD4 testing, and provide breast milk at enrolment; iv) the mother was willing to allow her infant to undergo study procedures, including full-course RV1 vaccination, phlebotomy at enrolment and 1 month post full RV1 vaccination, and presentation to clinic for collection of stool sample when infant had diarrhoea; and v) planned to remain in the area and was willing to come for scheduled visits for the duration of the study.

The University of Zambia Biomedical Research Ethics Committee, University of North Carolina at Chapel Hill Institutional Review Board and the Zambian Ministry of Health approved the study. This study did not require review by the Centers for Disease Control and Prevention institutional review board because only de-identified specimens were tested there.

The study was conducted in accordance with the principles of the Declaration of Helsinki and in compliance with good clinical practice guidelines; ClinicalTrials.gov registration number NCT 01886833.

### Clinic procedures

Enrolled mothers provided demographic and medical history information and provided expressed breast milk samples. Their delivery record was reviewed for information about their HIV status, and where it was not documented, they underwent HIV counselling, testing and CD4 count measurements.

Enrolled infants received a physical examination and a baseline serum sample was taken for rotvirus-specific IgA and IgG antibodies. The infant was then administered the full expanded programme on immunisation (EPI) vaccine regimen, which included the oral RV1 and polio vaccines, and parenteral pentavalent vaccines (containing diphtheria, tetanus, pertussis, haemophilus influenza, and hepatitis B). They were given an appointment card highlighting the next scheduled visit (one month post the second RV dose) and were escorted home by a research assistant in order to record the physical location of their residence, as there are no street addresses in the clinic catchment area. The mothers were asked to bring the child to the clinic anytime in case of illness of any kind. Arrangements for collection of stool samples were made, in the event the child developed diarrhoea.

### Laboratory procedures

Rotavirus-specific IgA in breast milk samples, and serum rotavirus specific-IgA and IgG were determined by an antibody capture ELISA assay as described in detail previously [27]. Briefly, microplate wells were coated with rabbit hyperimmune serum to rhesus rotavirus (RRV) and incubated with diluted RV1 strain or blotto (5% skim milk in phosphate-buffered saline [PBS]). After washing, breast milk and serum samples (1:20–1:20,480) that were serially diluted in diluent buffer (1% skim milk and 0.5% [v/v] of 10% polyoxyethylene ether W1 in PBS) were added to the wells, followed by biotin-conjugated goat antihuman IgA antibodies (KPL, Gaithersburg, Maryland). After incubation and washing, extravidin (Sigma, St. Louis, Missouri) was added to the wells and incubated, and then the reactions were developed with 3,3′,5,5′-tetramethylbenzidine (TMB; Sigma) and stopped with 1N hydrogen chloride. Optical density (OD) was determined at 450 nm with an enzyme immunoassay reader (MRX Revelation; Dynex Technologies, Chantilly, Virginia). IgA titres in breastmilk and serum were calculated as the reciprocal of the highest dilution that gave a mean OD greater than the cut-off value (3 standard deviations above the mean OD of the negative control serum wells). RV-specific IgG in serum samples was tested and analysed in the same manner as IgA except that 0.5% normal rabbit serum was added to the biotin-conjugated goat antihuman IgG antibody (KPL, Gaithersburg, Maryland) solution.

### Statistical analysis

Children were defined as having seroconverted when at 1 month post immunization, the rotavirus specific-IgA titre increased by four fold or greater compared with the titre recorded before the first dose of RV1. IgA titer ≥40 in serum was considered positive. IgA titer < 20 in serum is assigned a value of 1 for the calculation of GMTs. Pearson Chi-squared test was used to investigate the association of seroconversion and categorical factors (i.e. age, sex, season, maternal HIV status, and infant seropositivity at baseline) For breast milk rotavirus specific-IgA and infant serum rotavirus specific-IgG categorized as quartiles, Wilcoxon rank-sum test for trend was used.

Multivariable logistic regression was used to identify predictors of the odds of seroconversion. The antibody variables were transformed into log base 2 scales and modelled as a continuous covariate so that the effect would be interpreted as a doubling of antibody response. Season was considered a *priori* as an important confounder and was modelled as a categorical variable defined as 1 for dry (i.e. May to October at time of first dose) and 0 otherwise. In the multivariable model, covariates were included if the unadjusted P value for association with odds of seroconversion was 0.2 or less. Criteria for removing variables were set at P value < 0.2, and change in coefficients of variables is not more than 10%. Likelihood ratio test was used to investigate whether or not the effect of breast milk anti-rotavirus IgA on odds of seroconversion varies between seasons.

Geometric mean titres (GMT) and 95% confidence intervals of infant serum rotavirus specific-IgA post dose 2 and breast milk rotavirus specific-IgA were calculated for Wet and Dry seasons. With two dry seasons and one wet season, GMT’s of breastmilk were compared by season as well as infant serum IgA GMTs post vaccine dose 2 using a t-test. Wilcoxon rank-sum test was used to compare the median rotavirus specific-IgA titers in pre dose 1 breastmilk between non-seroconvertors and seroconvertors. Data were analysed using Stata 14 (StatCorp, College Station, Texas, USA).

## Results

**Fig 1:**
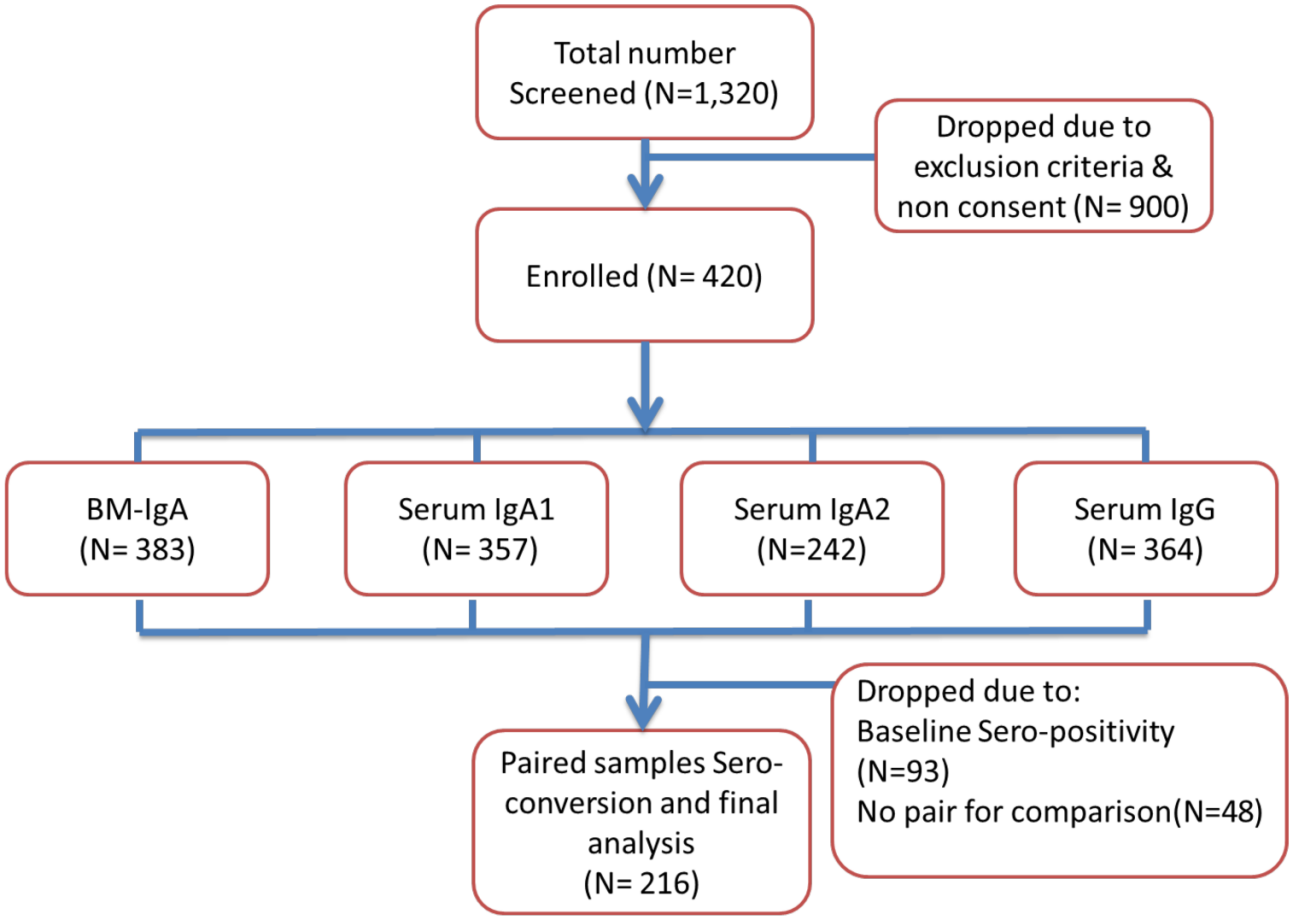
Study Participants Flow chart.

Of a total of 1320 infants and mothers were screened, 420 mother-infant pairs were successfully enrolled. The most common reason for declining to participate was unwillingness for the infant’s mother to have the infant’s blood drawn as shown in Fig 1.

Overall, the seroconversion frequency of IgA in the infants was 60.2% (130/216), while baseline seropositivity was 25.2% (90/357). Infants who were rotavirus specific-IgA seropositive at baseline were less likely to seroconvert compared to their seronegative counterparts (*P* = 0.04). There was no evidence of an association between maternal HIV status and seroconversion (*P* = 0.25). High titres of rotavirus specific-IgA in breast milk were associated with a lower frequency of seroconversion (Wilcoxon rank-sum test for trend z = -2.84; P<0.01) (Table 1).

**Table 1:** Percentage of infants seroconverted* by infant and maternal factors, in Lusaka.

Characteristics	Number of Infants (% of total)	Number (%) seroconversion	95% CI	Pearson Chi-square; P-value
**Age at first RV1** (Weeks)	n = 406	n = 211		**5.38; 0.07**
6 <7	249(61.3)	90(65.2)	[56.8, 72.8]	
7 <8	115(28.3)	24(47.1)	[33.7, 60.8]	
8–12	42(10.3)	12(54.6)	[33.6, 74]	
**Sex of infant**	n = 420	n = 216		0.03; 0.87
Female	221(52.6)	65 (59.6)	[50.1, 68.5]	
Male	199(47.4)	65 (60.7)	[51.1, 69.6]	
**Season**	n = 393	n = 216		0.41; 0.52
Wet	86(21.9)	13 (54.2)	[34.1, 72.9]	
Dry	307(78.1)	117 (60.9)	[53.8, 67.6]	
**Maternal HIV status**	n = 412	n = 211		1.34; 0.25
Negative	287(69.7)	88 (62.0)	[53.7, 69.6]	
Positive	125(30.3)	37 (53.6)	[41.7, 65.1]	
**Baseline infant serum IgA seropositivity**	n = 357	n = 216		**4.19; 0.04**
Seronegative	267(74.8)	105 (64)	[56.3, 71.1]	
Seropositive^1^	90(25.2)	25 (48.1)	[34.8, 61.7]	
**Breast-milk rotavirus specific-IgA (Quartiles)**	n = 383	n = 210		**-2.84; 0.01** ^2^
1 (1–80)	170(44.4)	65 (70.7)	[60.5, 79.1]	
2 (160)	84(21.9)	26 (54.2)	[39.9, 67.8]	
3 (320)	67(17.5)	19 (51.4)	[35.4, 67.1]	
4 (640–5120)	62(16.2)	15 (45.5)	[29.3, 62.7]	
**Infant serum rotavirus specific-IgG (Quartiles)**	n = 365	n = 215		**-1.88; 0.06** ^2^
1 (160–2560)	118(32.3)	53 (68)	[56.7, 77.4]	
2 (5120)	91(24.9)	32 (61.5)	[47.6, 73.8]	
3 (10240)	154(42.2)	44 (52.4)	[41.6, 62.9]	
4 (20480)	2(0.6)	1 (100)	-	
**Total**	**420 (100)**	**130 (60.2)**	**[53.5, 66.5]**	

* seroconversion = post immunization rotavirus specific-IgA titre increased ≥ four-fold compared with the titre recorded before the first dose of RV1

^1^ Seropositivity of IgA was defined as titer ≥ 1:40.

^2^ Wicoxon rank-sum test for trend

Significant differences are shown in bold.

**Fig 2:**
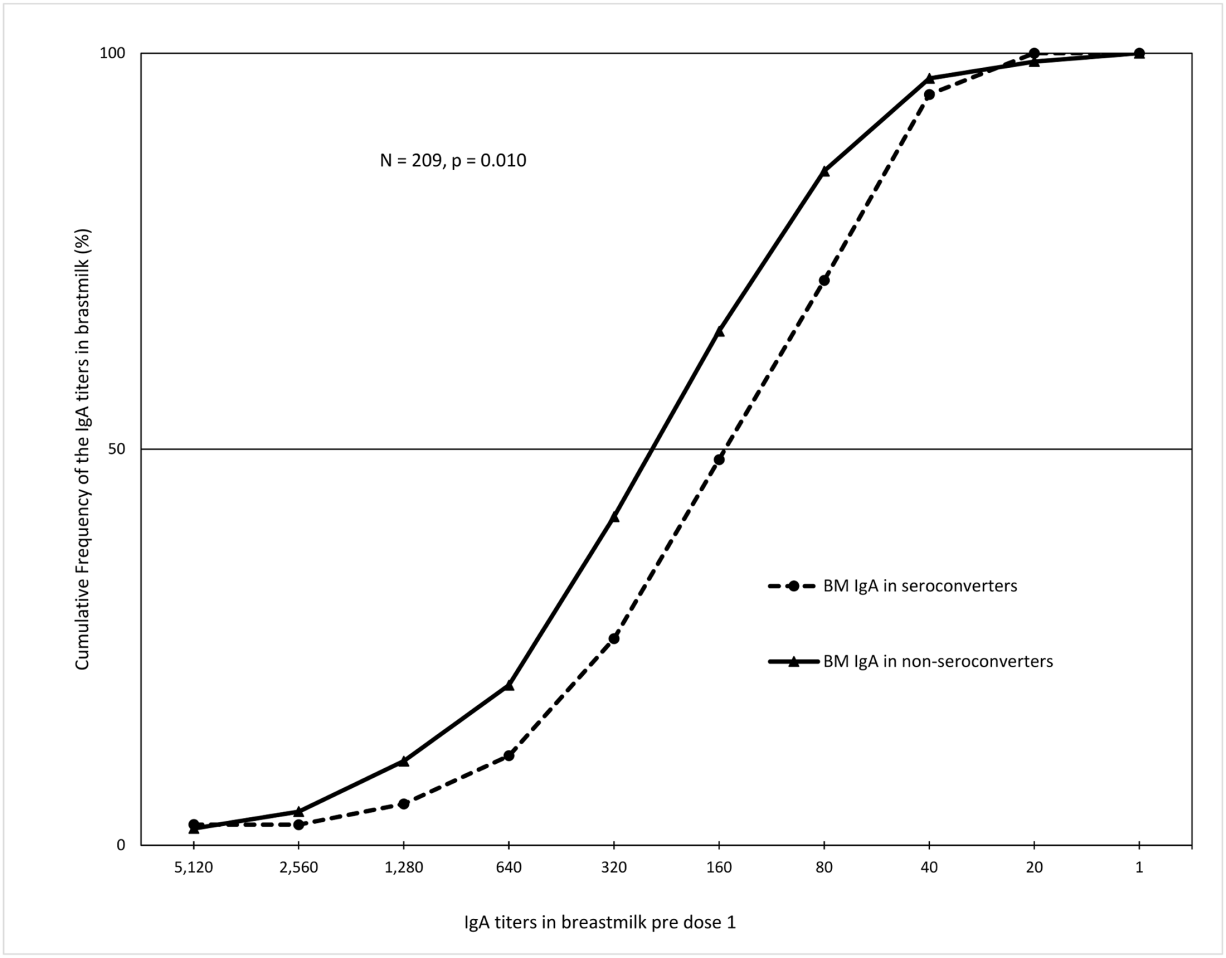
Cumulative frequency profiles of RV-IgA titres in pre dose 1 breastmilk of 216 mother-infant pairs by infant IgA seroconversion post dose 2 of rotavirus vaccine. Seroconversion was defined as four-fold increase in rotavirus-specific IgA titres in post dose 2 sera when compared to the corresponding pre-immunization sera.

When we plotted the cumulative frequency profiles of breastmilk rotavirus specific-IgA titres by infant IgA seroconversion (defined as four-fold increase in rotavirus-specific IgA titres in post dose 2 sera when compared to the corresponding pre-immunization sera), we observed a clear separation of the curves as shown in Fig 2. The median rotavirus specific-IgA titres in pre dose 1 breastmilk were significantly higher among non-seroconvertors compared with seroconvertors *(P* = 0.01). In a multivariable model with adjustment for seasonality, there was strong evidence of a 22% reduction in the odds of seroconversion, due to a two-fold increase in breast milk RV-specific IgA titres (Adjusted OR = 0.78; 95% CI = 0.66–0.92; P <0.01) (Table 2). When we analysed RV IgG levels in a univariable analysis, the doubling of infant RV IgG levels at baseline was associated with a 17% lower odds of seroconversion (Unadjusted OR = 0.83; *P* = 0.08).

**Table 2:** Maternal and infant factors independently associated with odds of seroconversion (N = 210).

Factors	Unadjusted OR (95% CI)	Unadjusted P-value	Adjusted OR (95%CI)	Adjusted P-value
**Age at first RV1(Weeks)**				
6 <7	ref	**0.07**		
7 <8	0.47(0.25,0.91)			
8–12	0.64(0.26,1.59)			
**Season**				
Wet	ref	0.52	ref	
Dry	1.32(0.56, 3.10)		2.37(0.91, 6.17)	**0.08**
**Maternal HIV status**				
Negative	ref	0.25		
Positive	0.71(0.40, 1.27)		-	
**Infant serum rotavirus specific-IgG** ^**1**^				
Values transformed to log_2_	0.83(0.68, 1.02)	**0.08**	-	
**Breastmilk rotavirus specific-IgA** ^**1**^				
Values transformed to log_2_	0.80(0.68, 0.94)	**0.01**	0.78(0.66, 0.92)	**<0.01**

We performed further analysis to examine the relationship between seasonality and immune response as a cause of rotavirus vaccine failure and found that dry cold seasons were significantly associated with higher IgA titers in breastmilk of mothers (Table 3). These higher IgA titers in breastmilk during the cold dry months (May to June) were negatively associated with IgA seroconversion to RV1 vaccination in infants. We observed an inverse trend between pre-dose 1 IgA seropositivity and seroconversion, but the associations were not significant.

**Table 3:** Rotavirus-specific IgA in Breastmilk and sera, and RV-IgA serconversion (SC), by seasons (N = 200).

		Breastmilk		Infant’s sera		
Season	n	IgA pre dose 1 GMT (95%CI)	IgA pre dose 1 positivity (%)	IgA pre dose 1 GMT (95%CI)	IgA post dose 2 GMT (95%CI)	IgA SC (%)
**Dry season 2013 (May 2013–Oct 2013)**	104	158 (122–204) (a)	27%	5 (4–8)	64 (37–110)	55% (d)
**Rainy season 2013–14 (Nov 2013–April 2014)**	31	85 (51–141) (b)	13%	5(3–10)	112 (50–252)	71% (e)
**Dry season 2014 (May 2014–Oct 2014)**	65	178 (140–227) (c)	20%	6 (3–9)	63 (31–130)	49% (f)
**P value**		p = 0.031 ^1^				p = 0.146 ^3^
		p = 0.010 ^2^				p = 0.051 ^4^

IgA seroconversion = post immunization rotavirus specific-IgA titre increased ≥ four-fold compared with the titre recorded before the first dose of RV1 Seropositivity of IgA was defined as titer ≥ 1:40.

^**1**^ t-test (a) vs (b),

^**2**^ t-test (b) vs (c),

^**3**^ X^2^-test (d) vs (e),

^4^ X^2^-test (e) vs (f).

## Discussion

In recent times, interference from maternal antibodies in breast milk has received particular attention as cause of rotavirus vaccine failure [21]. Several efforts have been made in randomised controlled trials of transient breastfeeding withholding around the time of vaccine administration. However, results from South Africa [27], Pakistan [28] and India [29] found no benefit from withholding breastfeeding around the time of immunisation. Although these studies did not find a benefit in RV vaccine immunogenicity with transient breastfeeding withholding, our results indicate a need to seek other interventions that may diminish potential breast milk interference of RV vaccine response.

There are key considerations that need to be made in light of these reports in order to justify continued research in this field. First, it remains debatable whether the hours of withholding breastfeeding implemented in these trials were sufficient to remove that potential interference. Second, in the case of the Asian trials, lower overall seroconversion rates were generally observed (16.6% in the withholding arm and 29.1% in the immediate breastfeeding arm in Pakistan; and 26.1% and 26.6% respectively in India), while our rate was much higher at 60.2%, similar to the 61% observed in South Africa [27]. Despite the fact that different assays with differing cut-offs for positivity were used in these trials, it does appear that breast milk effects could be variable depending on the background context and may still be the explanation for some of the unexplained results from various studies.

Our observations demonstrate the need to reconsider breast milk RV IgA carefully as a factor that could explain differential vaccine effectiveness rates between low, middle, and high-income settings. Whereas there was no evidence of an association between maternal HIV status and seroconversion (62% versus 54% respectively), infant seroconversion appeared to decrease with increasing levels of breast milk anti-RV IgA titers (71%, 54%, 51 and 46% respectively for each quartile).

Our proposed association between maternal immunity and infant seroconversion is further strengthened by our observations of seasonality trends. Our findings are consistent with existing epidemiological literature about RV transmission cycles: RV infections peak in the cool, dry season (April to July) and demonstrate a second, but smaller peak in the hot, dry season (October to November) [24–26, 28–31]. This study has shown consistent seasonal trends through GMT IgA titer in breast milk, reflecting the epidemiologic pattern when mothers would likely be getting infected. Further strengthening our argument, statistically significant differences in GMT tires have been seen by season among seroconvertors and non-seroconvertors, suggesting a negative relationship between breastmilk IgA titre and the post-dose 2 infant serum IgA titre across seasons. This observation requires further investigations into whether infants immunized in high RV transmission seasons yield lower seroconversion IgA titers, and the suggestion that there may be some interaction between seroconversion and season. It remains to be investigated whether the risk of clinical disease from RV is higher in infants with low vaccine induced IgA titres.

Maternally acquired IgG did show a trend to affect seroconversion upon interquartile baseline titre comparisons consistent with other studies [22]; however, in our study this association did not reach statistical significance (*p* = 0.06). Indeed IgG has been more strongly implicated in affecting seroconversion in other studies [22,32,33]. In univariate analysis, doubling infant IgG levels appeared to be associated with a 17% reduction in the odds of seroconversion but the effect diminished after accounting for other covariates. It is apparent that VP7 and VP4-specific immunoglobulin G (IgG) play an important protective role in natural infection and vaccine-mediated immunity, especially if their production is of sufficient magnitude for them to be exuded onto the mucosal surface where infection occurs [34–40]. These previously reported data support the theory that placental transfer of RV-specific IgG impacts vaccine effectiveness. In fact, higher antibody titre and higher rates of seroconversion and protection have been observed in older children without maternal antibody, in comparison to those of younger infants [38,41], suggesting maternal serum antibodies, in addition to those found in breast milk, attenuate response to vaccine [32,42,43].

Another important finding from our study is the relatively high rate of rate of pre vaccination IgA seropositives. At 25% baseline seropositivity, our findings suggest that a quarter of the infants are already exposed to rotavirus natural infection very early in life and these were less likely to serconvert after vaccination. With a borderline significance (P = 0.04), possibly because of our relatively small sample size, it remains to be concluded whether early natural exposure could also negatively influence vaccine seroconversion as previously observed in South Africa and Nicaragua [22, 27, 43].

The complexity and lack of understanding of seroconversion to vaccines and its meaning in terms of clinical protection still prevails at this point. Infant factors, including nutritional status, have been linked to RV diarrhoea. The relationship between malnutrition and RV vaccine efficacy remains a subject of interest for our on-going research. There are other suggestive reports that efficacy of oral vaccines is lower in poor hygiene settings where malnutrition is common [44, 45]. The actual effect of protein energy malnutrition and micronutrient deficiency on RV vaccine safety and efficacy is however not well understood [45,46].

Based on our findings, we conclude that there is a clear trend in the seasonal peaks of breast milk IgA and corresponding troughs in infant serum IgA response to RV vaccination. This inverse relationship suggests potential interference of maternal breast milk antibodies on seroconversion. The extent to which the effect of these maternal antibodies can be overcome by withholding breast milk for various periods of time around child immunisation remains to be demonstrated. Based on a number of studies in recent years [27, 32–34], It has become clear that transient withholding of breastfeeding does not appear to help. Given the logistic challenges of withholding and overwhelming benefits of breastfeeding, the scientific community seems to have largely decided to drop this transient intervention strategy. Nonetheless, we believe long-term breastfeeding has a damping effect on RV vaccines. Thus, there is need for further research into other interventions, strategies or approaches to explain this phenomenon. Lastly, it remains to be proved whether infants exposed to these high breast milk antibody titres are necessarily at higher risk of clinical disease.

As many LMICs seek to attain millennium development goals, reduction of child mortality through interventions that include “effective child vaccines” is an important strategy. Research such as ours, seeking to understand and explain challenges of achieving rotavirus vaccine impact is of great importance.

## Abbreviations

EIA: Enzyme Immunoassay
ELISA: Enzyme Linked Immunosorbent Assay
EPI: Expanded Programme on Immunisations
GE: Gastroenteritis
GCP: Good Clinical Practices
GSK: GlaxoSmithKline
GMT: Geometric Mean Titre
HCl: Hydrogen chloride
ID: Identification
IgA: Immunoglobulin A
IgG: Immunoglobulin G
HIV: Human Immuno Deficiency Virus
LMIC: Low Middle Income Country
NIH: National Institutes of Health
OPV: Oral Polio Vaccine
RV: Rotavirus
RV1: Monovalent Rotavirus vaccine (Rotarix™)
TB: Tuberculosis
